# Influence of social media on cosmetic facial surgeries among individuals in Kuwait: employing the theory of planned behavior

**DOI:** 10.3389/fdgth.2025.1546128

**Published:** 2025-04-24

**Authors:** Madhawi Alduosari, Thurayya Albuloshi, Ahmad Alsaber, Farah Al Saeed, Anwaar Alkandari, Amal Anbar, Bedour Alboloushi, Yasser Helmy

**Affiliations:** ^1^ Ministry of Health, Kuwait City, Kuwait; ^2^ Palliative Care Center, Ministry of Health, Kuwait City, Kuwait; ^3^ College of Business and Economics, American University of Kuwait, Salmiya, Kuwait; ^4^ Business and Management Department, Kuwait Technical College, Abu-Halifa, Kuwait; ^5^ Data Analytics Department, ASIA Consulting for Administrative, Kuwait City, Kuwait; ^6^ Department of Business and Management, Kuwait College of Science and Technology, Al Asimah, Kuwait; ^7^ Plastic and Reconstructive Surgery, Al-Azhar University, Cairo, Egypt

**Keywords:** cosmetic facial surgery, social media influence, youth attitudes, theory of planned behavior, body dysmorphic disorders

## Abstract

**Introduction:**

The increasing popularity of cosmetic facial surgeries among Kuwaiti youth has been significantly influenced by social media. Platforms such as Instagram and TikTok propagate beauty ideals that may lead to heightened interest in aesthetic procedures. This study investigates the impact of electronic word-of-mouth (e-WOM), content marketing (CM), and influencer marketing (IM) on attitudes (ATT), subjective norms (SN), perceived behavioral control (PBC), and intention (INT) to undergo cosmetic surgery, using the Theory of Planned Behavior (TPB) framework.

**Methods:**

A cross-sectional quantitative design was employed involving 730 participants (84.9% female), selected using convenience sampling across universities, clinics, and workplaces in Kuwait. A validated TPB-based questionnaire measured constructs such as ATT, SN, PBC, INT, health consciousness, and social media influences (e-WOM, CM, IM). Data were analyzed using Structural Equation Modeling (SEM) via SmartPLS. Reliability and validity were confirmed using Cronbach's alpha, composite reliability, average variance extracted (AVE), and discriminant validity.

**Results:**

Subjective norms significantly influenced perceived behavioral control (β = 0.336, *p* < 0.001), which in turn predicted intention to undergo cosmetic surgery (β = 0.316, *p* < 0.001). Attitude also positively influenced PBC (β = 0.298, *p* < 0.001). e-WOM had the strongest impact on ATT (β = 0.427, *p* < 0.001) and SN (β = 0.292, *p* < 0.001), followed by significant but smaller effects from CM and IM. The model demonstrated good fit, explaining 47.3% of the variance in INT.

**Discussion:**

Social media plays a central role in shaping cosmetic surgery intentions among Kuwaiti youth, with subjective norms being the strongest predictor. These findings underscore the importance of societal expectations, particularly in collectivist cultures. Regulatory frameworks and educational campaigns are recommended to address ethical marketing practices, enhance digital health literacy, and promote realistic beauty standards.

## 1 Introduction

Cosmetic surgery has witnessed a significant surge globally, with a nearly 50% increase in operations post-COVID-19, particularly in 2021 compared to the previous year (1). This trend is driven by a complex interplay of psychological, social, cultural, and situational factors, influenced by sex, age, race, culture, and nationality (2). In the Middle East, growing acceptance among the youth has contributed to a rising demand for cosmetic procedures, especially skin and nasal surgeries (3). However, there is ongoing debate regarding the primary drivers of cosmetic surgery decisions in this region, with some studies emphasizing cultural and familial expectations while others highlight the influence of social media (4). Despite this growing trend, there remains a lack of consensus regarding the exact drivers of cosmetic surgery decisions in the region. Some scholars argue that cultural and familial expectations play a more dominant role than social media influences, while others emphasize the increasing impact of digital platforms in normalizing cosmetic procedures. Social media usage has escalated worldwide, particularly during the COVID-19 pandemic, integrating itself into various aspects of daily life, including the aesthetics industry (5).

Platforms like Instagram, YouTube, and TikTok play a significant role in shaping beauty standards, often creating unrealistic ideals that impact self-esteem and body image (6). The accessibility of cosmetic surgery content on social media, from before-and-after images to influencer testimonials, has contributed to the normalization of cosmetic procedures as routine self-enhancements (7). Studies indicate a direct link between social media usage and the intention to undergo facial cosmetic surgery (8). Over 80% of practitioners now use social media platforms, such as Instagram, to showcase their work (9). There is also a direct link between a patient's intention to undergo facial cosmetic surgery and the use of social media (10). The growing accessibility of cosmetic surgery content on social media, ranging from before-and-after images to influencer testimonials, has contributed to the normalization of cosmetic procedures as part of routine self-enhancement.

Social media platforms that showcase trends and ideals to a worldwide audience, such as Instagram, YouTube, and TikTok, have tremendous impacts on beauty standards (11). These continuously changing standards frequently create unrealistic beauty standards that have detrimental effects on self-esteem and body image (12). For example, a study exploring middle-aged women's perceptions of cosmetic surgery revealed that body dissatisfaction, appearance investment, and aging anxiety are influenced by social media engagement (13). This makes it crucial to understand the motivations behind the increasing desire to pursue cosmetic modifications (14). Especially considering the increasing prevalence of post-surgery dissatisfaction despite heightened accessibility to cosmetic procedures (15).

Despite the growing body of research, a gap remains in understanding the exact impact of social media on cosmetic surgery, especially in specific cultural contexts (16). In the Middle East, studies show conflicting perspectives on whether social media or cultural barriers play a more dominant role in shaping attitudes toward cosmetic surgery (17). Additionally, legal and ethical considerations surrounding cosmetic surgery procedures vary across different countries, making it essential to analyze Kuwait's specific regulatory landscape.

Kuwait, with its distinct sociocultural landscape and high frequency of social media usage provides a suitable backdrop for exploring these dynamics (18). This study aims to investigate how social media influences the attitudes and decisions of individuals in Kuwait regarding cosmetic facial surgery using the Theory of Planned Behavior (TPB). As one of the most widely applied behavioural theories, TPB provides a robust framework for examining the determinants of human behaviour, making it particularly useful for understanding cosmetic surgery decisions in a socially and culturally nuanced context (19, 20).Cosmetic surgery is defined as a branch within plastic surgery centered on improving or repairing facial and body symmetry for patients desiring aesthetic improvements (21). Alternatively, cosmetic surgery can be defined as any elective procedure that involves altering the appearance to enhance physical appearance. Conversely (22), define cosmetic surgery as a combination of art and medical science, viewing cosmetic surgeons not just as medical professionals but also as artists. Facial plastic surgeries, a subset of cosmetic surgeries, focus on enhancing or restoring the appearance of a patient's facial area. These procedures encompass a wide range of interventions, including face and neck lifts, brow and forehead lifts, cosmetic blepharoplasty, rhinoplasties, facial implants, and injectables for various facial areas (23).

The global surge of cosmetic surgery has not escaped the Middle East. The perception of cosmetic procedures has become increasingly positive among the youth in the Middle East, resulting in a higher prevalence of skin and nasal surgeries (2). Western beauty ideals have driven many Middle Eastern women to seek cosmetic surgery (3).

While global trends indicate a growing acceptance of cosmetic surgery influenced by Western beauty ideals and social media exposure, Kuwait presents a unique cultural context that shapes cosmetic surgery decisions. In Kuwait, social norms, cultural expectations, and economic affluence significantly influence the demand for cosmetic procedures, particularly among young adults and women seeking facial enhancements such as rhinoplasty and Botox injections. A study conducted among college students in Kuwait revealed that media and peer influence play a pivotal role in shaping perceptions and decisions regarding cosmetic surgery, with female students exhibiting a higher propensity for cosmetic enhancements (24, 25). Additionally, social media platforms, including Instagram and Snapchat, contribute to the normalization of cosmetic procedures by promoting beauty standards that align with Western ideals (26).

Recent data suggests a steady rise in the popularity of cosmetic procedures in Kuwait, driven by cultural and social dynamics unique to the region. The prevalence of social media usage, combined with high economic affluence, further amplifies the demand for cosmetic enhancements (23). Despite the growing acceptance of cosmetic surgery, there remains a need for culturally sensitive public health interventions to address the influence of social media on body image perceptions (27). This study seeks to bridge the gap by examining how social media impacts subjective norms, attitudes, and perceived behavioral control regarding cosmetic facial surgery among Kuwaiti youth, using the TPB as the guiding framework (28).

The unique cultural context of Kuwait, characterized by high economic affluence and strong social media influence, shapes the landscape of cosmetic surgery acceptance. As young adults, particularly women, navigate these influences, it is essential to consider the implications of these trends for public health and societal norms surrounding beauty and self-image (29, 30).

According to a study by (4), plastic surgeons and dermatologists from Kuwait concur with a regional consensus on facial beauty standards. The consensus stated that “an oval or round face, full temples, and a well-defined jawline were considered signs of beauty, and facial anthropometry varied between Western and Middle Eastern women, as well as within the region” (4).

The decision to undergo cosmetic surgery is influenced by an intricate blend of psychological, social, cultural, and situational factors (5). Psychological aspects such as self-esteem, body image, and appearance anxiety are commonly associated with individuals who seek cosmetic procedures (29). Lower self-esteem and higher body dissatisfaction significantly impact patients’ decisions to pursue cosmetic surgery (30). A study by (6) titled “Demographic and Cultural Differences in the Acceptance and Pursuit of Cosmetic Surgery: A Systematic Literature Review” concludes that demographic factors such as sex, age, race, culture, and nationality significantly influence perceptions of cosmetic surgery (6).

One of the reoccurring factors that is often linked with cosmetic surgery is social media exposure. Social media also plays a significant role, particularly among young adults and women, by perpetuating ideals of perfection and increasing dissatisfaction with one's appearance (7). Moreover, the pervasive nature of social media has led to ethical concerns about the authenticity of cosmetic surgery marketing, with some studies suggesting that misleading content can contribute to unrealistic expectations among prospective patients.

This paper provides a comprehensive examination of the role of social media in the expansion of the facial cosmetic industry in Kuwait.

Social media has a significant effect on lifestyle choices including consumption patterns, social behavior, and health habits. The excessive usage of social media affects users’ daily habits, impacting their sleep routines and healthy consumption habits (31). Social media impacts have extended to the political landscape as well, The Internet and social media have been shown to impact political participation, voting behaviors, and attitudes towards the government (9). Additionally, the overuse of social media has been linked to negative body image. A study exploring middle-aged women's perception of cosmetic surgery revealed that body dissatisfaction, appearance investment, and aging anxiety are influenced by social media engagement (10). Social media platforms that showcase trends and ideals to a worldwide audience, such as Instagram, YouTube, and TikTok, have a tremendous impact on beauty standards. These continuously changing standards frequently create unrealistic beauty standards that have a detrimental effect on one's self-esteem and body image (11).

This paper provides a comprehensive examination of the role of social media in the expansion of the facial cosmetic industry in Kuwait. Beyond understanding individual motivations, this study also aims to provide insights into regulatory and ethical implications, bridging the gap between digital influence and healthcare policy considerations in the context of cosmetic surgery.

### 1.1 Problem statement

A rise in dissatisfaction post-surgery has accompanied the increase in cosmetic surgery procedures (12). Additionally, a study exploring the reasons behind undergoing cosmetic surgery revealed that women who opt for these procedures are more likely to undergo further surgeries (32). Specifically, women who have had rhinoplasties are 15% more likely to opt for a revision rhinoplasty (13). While the rapid growth of the cosmetic industry has garnered significant research interest, particularly regarding the influence of social media on the decision to undergo cosmetic surgery, there remains a gap in the literature on the exact impact of social media (14). Kuwait has been chosen as the main context of this study due to its unique sociocultural landscape and the growing popularity of cosmetic procedures among its youth. These factors make Kuwait an ideal setting for exploring the influence of social media on cosmetic surgery decisions. This study aims to identify the underlying factors driving the cosmetic surgery trend particularly the role of social media. There is no denying that Kuwait mirrors the global trend of widespread social media usage.

In Kuwait, social media spans various domains, including education, government communication, and business, reflecting its integral role in the country's digital landscape (15). Additionally, there is a significant prevalence of cosmetic surgery in Kuwait, influenced by various factors such as media influence, social acceptance, and advancements in medical technology (16). Kuwait has been chosen as the context of this study due to its unique reliance on social media and the observable rise in elective cosmetic surgery.

### 1.2 Research purpose

#### 1.2.1 Study objective

This study aims to identify the factors that influence cosmetic surgery intentions among Kuwaiti youth, particularly examining the role of social media, subjective norms, attitudes, and perceived behavioral control. By focusing on ‘what’ factors contribute to these intentions, the study provides a comprehensive understanding of the underlying drivers of cosmetic surgery decisions.

### 1.3 Importance of the study

This study is pivotal because it investigates the complex relationship between social media use and the rising trend of cosmetic face operations in Kuwait. Given the explosive expansion of the cosmetics business and the pervasive impact of social media, it is critical to comprehend the underlying causes that motivate people—especially young people—to pursue cosmetic modifications (17). By examining the precise influence of social media on the decision-making process associated with cosmetic facial operations, this study closes a significant gap in the literature.

The overall motivations for having cosmetic treatments have been studied in previous studies, but the immediate impact of social media has not received as much attention (33). The study's concentration on this field yields insightful information that can direct future investigations and the formulation of policies.

Kuwait provides a suitable backdrop for this study because of its distinct sociocultural landscape and high frequency of social media usage (18). The results can provide a better understanding of how regional patterns in cosmetic surgery are shaped by the interaction of cultural and digital variables. This cultural viewpoint adds to the global conversation on cosmetic surgery by emphasizing the need to treat the phenomena with context-specific strategies. The study's conclusions have applications for a range of stakeholders, such as marketers, legislators, and healthcare professionals. Through the identification of significant factors including Subjective Norms, Content Marketing, and Perceived Behavioral Control, the study offers practical insights that might guide initiatives aimed at controlling the growing demand for cosmetic surgery.

Health care professionals can create strategies aimed at proper screening of patients in order to reduce dissatisfaction post-surgery. On the other hand, marketers can employ ethical methods in their promotion of cosmetic procedures. or negative mental effects aimed at proper addressing.

Employing the TPB, this study contributes to the theoretical understanding of cosmetic surgery intentions. By integrating TPB with machine learning techniques, the study demonstrates a comprehensive methodological approach that enhances the predictive accuracy of behavioral intentions. The study sheds light on gender disparities and the influence of social norms on cosmetic surgery decisions. By examining how subjective norms and social media pressures impact different genders, the research provides nuanced insights that can inform gender-sensitive policies and campaigns. This focus on social norms also highlights the role of societal expectations in shaping individual behaviors, and emphasizes the need for integrating strategies that address both individual and collective influences.

## 2 Literature review

### 2.1 Theoretical framework: theory of planned behavior (TPB)

Behavioral theories uncover significant information on human behavior and why humans behave the way they do in individual, social, and environmental contexts (20).

The theory of planned behavior (TBD) is often employed to understand the determinants of intention to conduct a certain action (34). It is one of the most commonly used theories in predicting behaviors due to its focus on behavior determinants. Its popularity is also attributed to the substantial empirical support it has garnered (34). According to TBD, human behavior is directed by three main factors: attitude toward the behavior, subjective norm, and perceived behavioral control (35). Attitude refers to the individual's positive or negative evaluation of conducting a behavior (36). In other words, a person's reflection of the degree of favorability of the behavior in question (37).

It encompasses beliefs about the outcomes of the behavior and the evaluations of these outcomes. For instance, in a study that utilized the TPB to understand the behavior of individuals with disabilities in the context of tourism, it was concluded that a positive attitude towards travel substantially influenced their travel intentions (38). Another compelling example of how attitudes influence behavior is the Islamic banking industry. Specifically, a positive attitude towards utilizing Islamic banking services significantly enhances the likelihood of individuals choosing these services (39).

Another dimension includes social norms, which explain how social pressure impacts an individual's decision to engage in certain behaviors. Individuals are influenced by their perceptions of societal expectations. A person is more likely to act if they believe it is expected of them by people who hold significance in their lives. A recent study on vaccination revealed that individuals’ decisions to undergo COVID-19 vaccination were substantially influenced by their peers and societal expectations (40). A study aimed at predicting behavior related to the purchase of health insurance, in Malaysia, serves as a great example on how social norms influence individuals’ decisions. It concluded that societal expectations had a noticeable positive effect on the intention to purchase health insurance (41).

Finally, PBC refers to an individual's perception of the ease or difficulty in performing a task. This perception is influenced by factors such as the presence or absence of obstacles, the individual's capabilities, and the amount of effort required to complete the task (42). A study that employed the TPB to examine behavior related to fitness demonstrated that individuals who had confidence in their ability to exercise were more likely to engage in physical activity. This confidence, often referred to as perceived behavioral control, significantly influences their exercise intentions and actions (34). Based on the theory of planned behavior, people are more likely to carry out their intended actions and express intentions that match their attitudes and the influence of social norms when they have a strong sense of perceived behavioral control (19).

The theory of planned behavior is deployed in this study to investigate the role of social media in the intention to undergo facial cosmetic surgery in Kuwait.

### 2.2 Cosmetic facial surgeries: trends and influences

The impact of COVID-19 on the global spike in cosmetic surgeries has been significant. A study conducted to investigate whether COVID-19 affected the facial plastic surgery industry globally revealed a substantial rise in interest in facial aesthetic procedures post-COVID-19 (43).

Amongst the plethora of reasons behind this surge, a prominent factor was the shift from in-office work to working from home which allowed patients privacy and sufficient recovery time without the disruption of professional obligations (44). Above all, the most noted culprit of the amplification of facial cosmetic surgery is attributed to exposure to social media. During COVID-19 social media usage escalated amongst users globally. There is a direct link between patient's intention to conduct facial cosmetic surgery and their usage of social media (44).

In contrast, in a study examining the Indian cosmetic surgery industry, it was found that the increased demand for facial plastic surgery is attributed to a shift in patients’ attitudes towards nonsurgical aesthetic procedures, as well as the modernization of techniques used by surgeons (45).

Above all, the most noted culprit of the amplification of facial cosmetic surgery is the rise in exposure to social media. During COVID-19 social media usage escalated amongst users globally (46). There is a direct link between patients’ intentions to conduct facial cosmetic surgery and their social media usage. Patients who used more social media were more likely to undergo facial procedures (44). While the global impact of COVID-19 had a considerable impact on cosmetic surgery intentions it is vital to also consider the cultural, social, and psychological aspects.

Cultural acceptance majorly dictates intentions when it comes to cosmetic surgery (47). The globalization of beauty standards has led women across the world to opt for the surgical route to meet those standards (48). Societal emphasis on beauty ideals affects the pursuit of cosmetic procedures (49). A study exploring the reasons behind women's decisions to undergo cosmetic procedures revealed that most Women who conducted surgery believed the change in their appearance would improve their social stature and acceptance into society (50). Bascarane et al. (51) concur that women suffering from psychiatric disorders are more likely to undergo cosmetic surgery. According to (51), “The prevalence of psychiatric disorder in patients undergoing cosmetic surgery was 4%–57% for body dysmorphic disorder (BDD); the corresponding figures for depression, anxiety, and personality disorder were 4.8%–25.8%, 10.8%–22%, and 0%–53%, respectively” (51).

Whereas patients with mental disorders are more likely to undergo cosmetic procedures, social media users are more prone to developing mental disorders. This cyclical relationship is further explored in this research paper (52).

### 2.3 Social media and cosmetic surgery

Social media has infiltrated every facet of human existence. From business utilizations to personal interactions and even medical applications, the wide-range use of social media has woven social media platforms into every crevice of daily life (53). This phenomenon has not escaped the cosmetic industry. Social media is among the most influential factors impacting the cosmetic industry. The influence of social media in the realm of cosmetics encompasses both practitioners and patients (54).

Conversely, exposure to post-op images of cosmetic surgery patients increases viewers’ desire to undergo surgery by creating dissatisfaction with their appearance upon compared with the post-op photos (27). From another angle, social media plays a role in shaping beauty standards. Beauty influencers on social media platforms such as Instagram and TikTok shape public perception by promoting specific beauty ideals (55). Social media users often compare themselves to the influencers they follow, which can lead to dissatisfaction with their appearances (56).

Another factor resulting in appearance dissatisfaction amongst social media users is the incorporation of aesthetic filters on social media platforms. Social media platforms like Snapchat have introduced technological filters that enhance a user's appearance to align with trending beauty standards (57). Furthermore, this trend has led to the rise of beauty-enhancing apps such as FaceApp, which inevitably increase users’ dissatisfaction with their natural appearance and result in higher intentions to pursue plastic surgery (58). The more users are exposed to filters the more likely they are to conduct plastic surgery (59). Another significant concept in the context of social media is electronic word of mouth (e-WOM). Electronic Word of Mouth (e-WOM) represents a contemporary version of traditional word-of-mouth communication, greatly enhanced by the reach of the internet and social media (60). Research suggests that sharing information, opinions, and reviews online significantly impacts consumer behavior and decision-making (61).

E-WOM facilitates the widespread distribution of information among social media users (62). This concept is crucial when it comes to cosmetic surgery, as one determinant of cosmetic surgery intentions is the globalization of beauty standards, which has resulted from e-WOM.

### 2.4 A local context

Social media usage has seen an upward growth in recent years in the Middle East (63). The surge of social media in the Middle East and North Africa (MENA) region has been spearheaded by the youth in the region (64). Platforms such as Snapchat and Instagram have been linked to increased depression risk among youth (65). A study conducted in Saudi Arabia revealed “that 42.6% of participants cited surgeon self-advertising and 38.0% cited better selfies as influencing factors in their decision to undergo cosmetic surgery” (66). Mirroring the global context, there is a link between the rise in engagement on social media and the upsurge in the demand for plastic surgery in the Middle East (26).

Nevertheless, the Middle Eastern region is peculiar when it comes to cultural impacts on decision-making. Family dynamics, religious and spiritual needs, and social values are amongst the factor's the region's cultural landscape (67).

A study conducted to explore attitudes toward plastic surgery in Kuwait revealed that, despite the cultural barriers that contribute to hesitation about undergoing plastic surgery, social media-instigated peer pressure has driven many Kuwaiti youths to seek plastic surgery (16).

Furthermore, while the literature in the Middle East presents conflicting perspectives on the influence of cultural barriers vs. social media pressure regarding plastic surgery, it is evident that the beauty and cosmetics industry in the region has seen substantial growth (68). This indicates that more attention should be paid to this matter.

## 3 Methodology

### 3.1 Study design

This was a cross-sectional study of people of all ages living in Kuwait. A convenience sampling methodology was used to recruit participants at various Universities, Hospitals, Clinics, and workplaces, including business owners, government employees, private sector employees, retirees, students, and those currently unemployed, representing a diverse range of work and life situations in Kuwait between July 2023 and September 2024. Inclusion criteria included Kuwaiti nationals aged 18 and above who consented to participate in the study, while exclusion criteria involved non-Kuwaiti nationals and individuals under 18 years of age. Recruitment continued until 730 participants were enrolled. A power calculation determined that a minimum sample size of 700 participants was required to achieve sufficient statistical power to detect significant effects at a 95% confidence level with an effect size of 0.3.

Data was collected through an online questionnaire distributed among participants via email. Participants completed a validated questionnaire based on the TPB to enhance the predictive accuracy of behavioural intentions. According to TBD, human behaviour is directed by three main factors: attitude toward the behaviour, subjective norms (SN), and perceived behavioral control (PBC) (35). Attitude refers to an individual's positive or negative evaluation of their behavior (36). In other words, a person reflects on the degree of favorability of the behaviour in question (37). Attitudes have been shown to influence behavioural intentions (38). Subjective norms refer to the perceived social pressure from influential individuals, like family, friends, and social media influencers, to engage in or avoid a particular behavior, in this case, cosmetic facial surgeries. PBC refers to an individual's perception of the ease or difficulty of performing a task. This perception is influenced by factors such as the presence or absence of obstacles, an individual's capabilities, and the amount of effort required to complete the task (42). Based on the TPB, people are more likely to carry out their intended actions and express intentions that match their attitudes and the influence of SN when they have a strong sense of PBC (20).

The questionnaire, which took approximately 30 minutes to complete, included 30 questions. Questions included those related to baseline characteristics (i.e., age, sex, occupation, education level, marital status, nationality), assessing intention to get cosmetic facial surgery (INT), attitudes towards cosmetic facial surgery (ATT), subjective norms (SN), perceived behavioural control (PBC), health consciousness, and social media use [i.e., electronic word of mouth (e-WOM), influencer marketing (IM), content marketing (CM), and information search]. Electronic Word of Mouth (e-WOM) represents a contemporary version of traditional word-of-mouth communication that is greatly enhanced by the reach of the Internet and social media (60). E-WOM facilitates the widespread distribution of information among social media users (62). Participants were asked to rate their agreement with various statements on a Likert scale.

To ensure validity and reliability, the questionnaire was adapted from well-established scales used in past research, and it was subjected to a pilot test. The results of the pilot test were analyzed to confirm the reliability (e.g., using Cronbach's alpha) and the validity of the constructs, ensuring that the questions accurately measured the intended concepts.

The questionnaire was first developed in English and designed in a Western cultural frame and context, it was translated to Arabic and back-translated into English by two processional independent translators. This procedure is considered a quality assurance method to compare the back-translated version with the original version to ensure that the items remain in the same original context.

### 3.2 Ethical considerations

This study adhered to the highest ethical standards in human research. Ethical approval was obtained from the Ethics Committee of Kuwait Technical College (K-Tech) before initiating the study. The research was conducted following the ethical guidelines of the Declaration of Helsinki, ensuring respect for participants’ autonomy, confidentiality, and informed consent.

All participants were informed about the purpose of the study, the procedures involved, their right to withdraw at any time without penalty, and the measures taken to protect their privacy and confidentiality. Written informed consent was obtained from each participant prior to data collection. The data collected was anonymized and securely stored, ensuring that no personally identifiable information was accessible to unauthorized individuals.

### 3.3 Data analysis

Descriptive statistics were calculated to summarize the demographic characteristics and responses for each questionnaire construct.

We used Partial Least Squares (PLS)—Structural Equation Modeling (SEM). SEM is a robust method for simultaneously analyzing multiple relationships. Unlike regression-based techniques, Partial Least Squares (PLS) evaluates numerous latent constructs using a variety of manifest variables (69). This approach involves a two-step process: first, assessing the outer measurement model, followed by evaluating the inner measurement model, also known as the structural model (70).

Indicator reliability, construct reliability, convergent validity, and discriminant validity were confirmed through various metrics, including loadings, composite reliability, Average Variance Extracted (AVE), and the Fornell-Larcker criterion as recommended by (71). Reliability is a crucial characteristic of all indicators. Indicator reliability was assessed by examining the item loadings (72). A reliability value greater than 0.6 is generally accepted as an indication of indicator reliability. Composite reliability and Cronbach's alpha were used to assess the reliability of the underlying construct in SEM. A construct is considered reliable with a value of composite reliability and Cronbach's alpha greater than 0.7. Further, to assess convergent validity, the AVE was utilized, which represents the proportion of variance captured by the construct in relation to measurement error. The assessment of convergent validity requires that the AVE be greater than 0.5, ensuring that the construct explains more than half of the variance in its indicators (71, 73).

Collinearity occurs when the predictor variables are highly correlated, potentially leading to inflated standard errors and unreliable estimates. Collinearity was checked using the Variance Inflation Factor (VIF) and results were considered acceptable when VIF value is below the threshold value of 10 (48).

Discriminant validity was assessed to ensure that the measures of one construct did not correlate with those of another (74). The common method for evaluating discriminant validity are Fornell and Larcker's (1981) criteria The Fornell and Larcker (1981) criterion (75) involves comparing the square root of each construct's AVE with its bivariate correlations with all other constructs (75). To confirm discriminant validity, the square root of each construct's AVE should exceed its bivariate correlations with other constructs (74). However, this strategies have been found inadequate for assessing discriminant validity. Consequently, a more precise criterion called the heterotrait–monotrait ratio of correlations (HTMT) has been introduced in the literature (76). The HTMT measures the average correlation between the indicators of different constructs relative to the average correlation between the indicators of the same construct (76). To confirm discriminant validity, the HTMT value between the two constructs should be less than 0.90.

After validating the measurement model, the next phase examines the structural model, also known as the inner model. This step involves a thorough analysis of the relationships between the constructs within the model (77).

In this study, direct and indirect effects were determined using Partial Least Squares Structural Equation Modeling (PLS-SEM) with the SmartPLS software. Direct effects represent the relationship between two constructs connected by a single path, calculated through path coefficients. Indirect effects occur when the relationship between two constructs is mediated by one or more intervening variables. Both direct and indirect effects were assessed for statistical significance using the bootstrapping technique, where 5,000 resamples were generated to obtain T-statistics and *p*-values. This method allows for the estimation of the stability and significance of the model's relationships, ensuring that both direct and indirect paths are supported by robust statistical evidence. *P*-values <0.05 were considered statistically significant.

## 4 Results

The demographic characteristics of the participants are presented in Table 1. The study sample was comprised of 730 participants. Most were female (84.9%), 18–34 years old (66.7%), single (56.8%), Kuwaiti nationals (91.1%), and held a bachelor's degree (58.6%). A large proportion were students (48.8%) and government employees (32.7%).

**Table 1:** Demographic characteristics.

Demographic	Overall (*N* = 730)	%
Gender		
Female	620	84.9%
Male	110	15.1%
Age group		
<18	35	4.8%
18–24	342	46.8%
25–34	145	19.9%
35–44	107	14.7%
45–54	69	9.5%
55–64	28	3.8%
65+	4	0.5%
Nationality		
Kuwaiti	665	91.1%
Non Kuwaiti	65	8.9%
Occupation		
Business owner	27	3.7%
Government employee	239	32.7%
Private sector employee	38	5.2%
Retired	41	5.6%
Student	356	48.8%
Unemployed	29	4.0%
Education		
Bachelor	428	58.6%
Diploma	123	16.8%
High school	140	19.2%
Master	25	3.4%
Phd	14	1.9%
Martial state		
Divorced	42	5.8%
Married	260	35.6%
Single	415	56.8%
Widowed	13	1.8%

**Figure 1:**
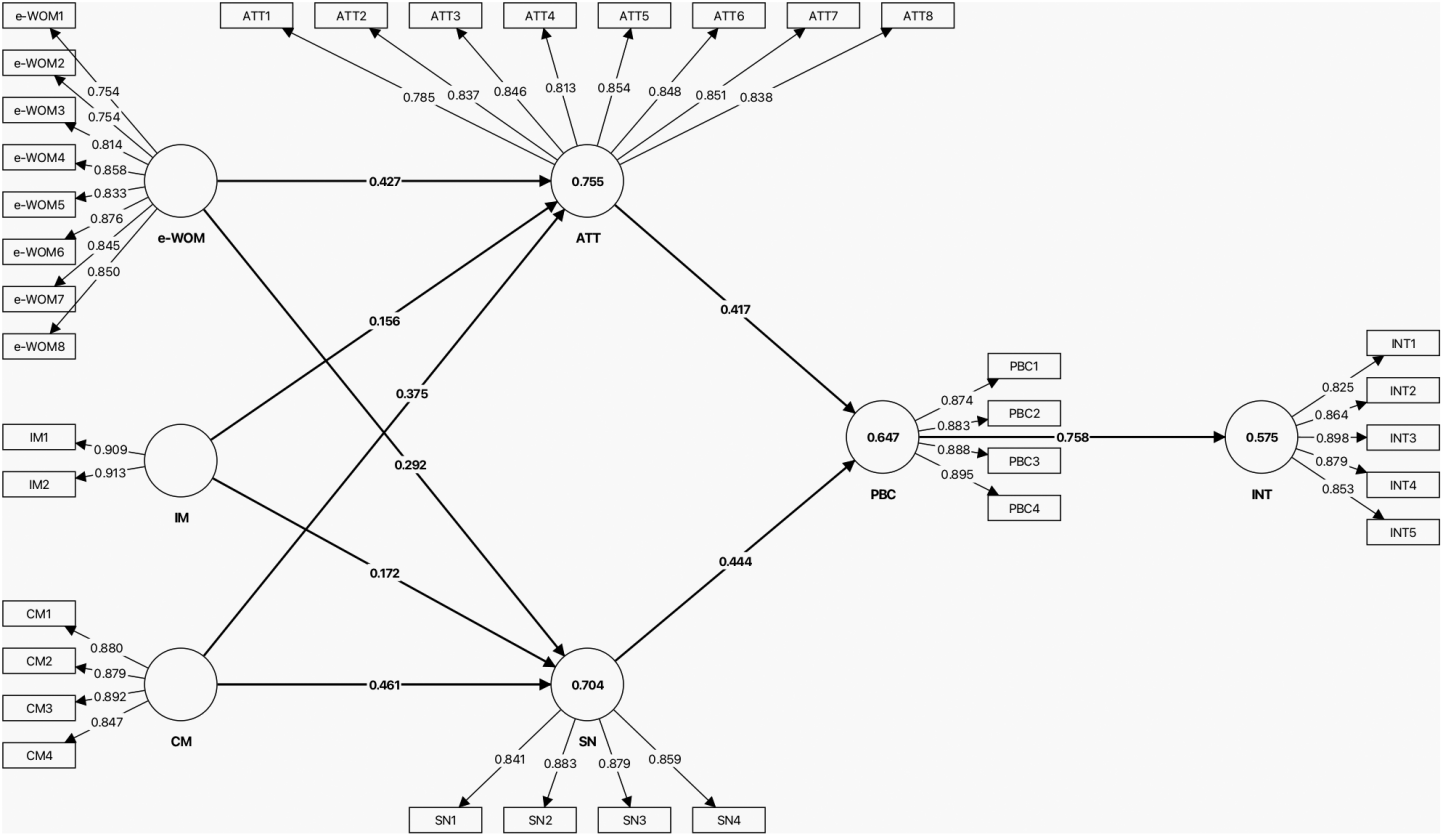
Model node diagram with loading.

Figure 1 illustrates the basic node diagram for the SEM along with the associated loadings. The nodes represent the key constructs, such as e-WOM, IM, CM, ATT, SN, PBC and INT. The loading values indicate the strength of the relationship between each indicator and its respective construct. These values were determined using Partial Least Squares (PLS) Structural Equation Modeling (SEM), a robust analytical technique that simultaneously analyzes multiple relationships between latent variables. This allows for a comprehensive understanding of the constructs aind their indicators.

The outer loadings for all items exceeded 0.60, indicating that the corresponding indicator strongly reflects its associated latent construct (71). The values for both Cronbach's alpha and composite reliability exceed 0.70 (Table 2), confirming that the constructs were reliable. The value of Average Variance Extracted (AVE) for all constructs was more than 0.5, confirming the convergent validity of the constructs. The VIF values ranged from 1.767 to 3.583, indicating no multicollinearity issues (Table 2). The results presented in Table 3 show that the square root of each construct's AVE exceeds its bivariate correlations with other constructs. As shown in Table 4, the HTMT values for all constructs were below 0.90, confirming discriminant validity.

**Table 2:** Outer model summary table for PLS-SEM.

Construct	Items	Loading	VIF	Cronbach's alpha	Composite reliability (rho_a)	Composite reliability (rho_c)	Average variance extracted (AVE)
	ATT1	0.785	2.218	0.937	0.938	0.948	0.696
	ATT2	0.837	2.947				
	ATT3	0.846	3.043				
	ATT4	0.813	2.654				
	ATT5	0.854	3.018				
	ATT6	0.848	3.022				
	ATT7	0.851	3.542				
	ATT8	0.838	2.968				
	CM1	0.880	2.627	0.897	0.898	0.929	0.765
	CM2	0.879	2.597				
	CM3	0.892	2.892				
	CM4	0.847	2.127				
	IM1	0.909	1.767	0.794	0.795	0.907	0.829
	IM2	0.913	1.767				
	INT1	0.825	2.526	0.915	0.917	0.936	0.747
	INT2	0.864	3.000				
	INT3	0.898	3.282				
	INT4	0.879	2.999				
	INT5	0.853	2.693				
	PBC1	0.874	2.545	0.908	0.909	0.935	0.783
	PBC2	0.883	2.687				
	PBC3	0.888	2.742				
	PBC4	0.895	2.853				
	SN1	0.841	2.186	0.889	0.890	0.923	0.750
	SN2	0.883	2.579				
	SN3	0.879	2.561				
	SN4	0.859	2.254				
	e-WOM1	0.754	1.976	0.932	0.934	0.944	0.679
	e-WOM2	0.754	1.924				
	e-WOM3	0.814	2.426				
	e-WOM4	0.858	3.009				
	e-WOM5	0.833	2.680				
	e-WOM6	0.876	3.583				
	e-WOM7	0.845	2.748				
	e-WOM8	0.850	2.921				

**Table 3:** Fornell-Larcker criterion results.

Construct	ATT	CM	IM	INT	PBC	SN	e-WOM
ATT	0.834						
CM	0.809	0.875					
IM	0.673	0.674	0.911				
INT	0.794	0.788	0.631	0.864			
PBC	0.748	0.776	0.575	0.758	0.885		
SN	0.747	0.802	0.663	0.770	0.755	0.866	
e-WOM	0.812	0.771	0.619	0.745	0.743	0.754	0.824

**Table 4:** HTMT ration results.

Construct	ATT	CM	IM	INT	PBC	SN	e-WOM
ATT							
CM	0.881						
IM	0.780	0.798					
INT	0.857	0.870	0.740				
PBC	0.809	0.858	0.678	0.830			
SN	0.818	0.897	0.789	0.851	0.838		
e-WOM	0.869	0.842	0.719	0.806	0.808	0.827	

The results of the structural model analysis provide strong support for all the hypothesized relationships (Table 5). These findings confirm the validity of the proposed model and all the hypothesized relationships.

**Table 5:** Bootstrap results for the inner model regression paths (direct effect).

Hypothesis	Original sample (O)	Standard deviation (STDEV)	T statistics (|O/STDEV|)	*P* values	Decision
ATT -> PBC	0.417	0.043	9.681	<0.001	Supported
CM -> ATT	0.375	0.043	8.783	<0.001	Supported
CM -> SN	0.461	0.047	9.777	<0.001	Supported
IM -> ATT	0.156	0.035	4.526	<0.001	Supported
IM -> SN	0.172	0.037	4.684	<0.001	Supported
PBC -> INT	0.758	0.022	35.185	<0.001	Supported
SN -> PBC	0.444	0.044	10.096	<0.001	Supported
e-WOM -> ATT	0.427	0.040	10.618	<0.001	Supported
e-WOM -> SN	0.292	0.041	7.192	<0.001	Supported

Structural model analysis revealed significant indirect effects, further supporting the hypothesized relationships within the proposed model (Table 6). These findings confirm the hypothesized indirect relationships, illustrating the complex interactions among the constructs.

**Table 6:** Bootstrap results for the inner model regression paths (mediation analysis or indirect effect).

Hypothesis	Original sample (O)	Standard deviation (STDEV)	T statistics (|O/STDEV|)	*P* values	Decision
IM -> SN -> PBC	0.076	0.017	4.577	<0.001	Supported
SN -> PBC -> INT	0.336	0.034	9.802	<0.001	Supported
CM -> ATT -> PBC	0.156	0.025	6.162	<0.001	Supported
e-WOM -> SN -> PBC	0.130	0.022	5.797	<0.001	Supported
IM -> ATT -> PBC	0.065	0.015	4.410	<0.001	Supported
e-WOM -> ATT -> PBC	0.178	0.026	6.906	<0.001	Supported
CM -> SN -> PBC -> INT	0.155	0.025	6.294	<0.001	Supported
e-WOM -> ATT -> PBC -> INT	0.135	0.021	6.473	<0.001	Supported
IM -> SN -> PBC -> INT	0.058	0.013	4.543	<0.001	Supported
e-WOM -> SN -> PBC -> INT	0.098	0.017	5.761	<0.001	Supported
CM -> ATT -> PBC -> INT	0.119	0.020	5.875	<0.001	Supported
IM -> ATT -> PBC -> INT	0.049	0.011	4.309	<0.001	Supported
ATT -> PBC -> INT	0.316	0.036	8.724	<0.001	Supported
CM -> SN -> PBC	0.204	0.032	6.428	<0.001	Supported

## 5 Discussion

The influence of social media on cosmetic surgery intentions, particularly in the context of Kuwait, is a multifaceted phenomenon that aligns closely with the TPB. This theory posits that subjective norms, attitudes, and perceived behavioral control are critical predictors of an individual's intentions to engage in specific behaviors, including cosmetic surgery (27). The study indicates that social media significantly shapes these predictors, particularly through electronic word of mouth (e-WOM) and content marketing, which foster favorable attitudes towards cosmetic procedures (27). Research has shown that exposure to positive narratives and testimonials about cosmetic surgery on platforms like Instagram and TikTok can enhance individuals’ willingness to undergo such procedures, reinforcing the normalization of cosmetic surgery within societal beauty standards (27, 78).

In the cultural context of Kuwait, the impact of social media is particularly pronounced among the youth, who are avid users of these platforms. The study reveals that Kuwaiti individuals are increasingly influenced by Western beauty ideals propagated through social media, which often showcases curated images that set unrealistic standards of beauty. This cultural shift is significant, as it reflects a broader trend where social media serves as a conduit for Western aesthetics, thereby reshaping local perceptions of beauty and self-image. The collectivist nature of Kuwaiti society further amplifies this influence, as individuals often seek social approval from family and peers, making subjective norms a powerful predictor of their intentions to pursue cosmetic surgery (27).

Moreover, the role of influencers and content marketing cannot be overstated. Participants in the study reported being significantly influenced by social media content that highlights positive experiences with cosmetic procedures, which in turn increases their propensity to consider surgery (27). This aligns with findings from other research that emphasizes how influencers often conform to and promote a narrow definition of beauty, which can lead to body dissatisfaction and increased desire for cosmetic enhancements among their followers (27, 79). The psychological implications of this phenomenon are concerning, as the pressure to conform to these beauty standards can exacerbate body image issues, particularly among vulnerable populations (80).

This study's findings align with global research indicating that social media plays a pivotal role in influencing cosmetic surgery intentions through mechanisms like electronic word-of-mouth (e-WOM) and influencer marketing (81). Similar to findings from Saudi Arabia and Iran, Kuwaiti youth are heavily influenced by social media's portrayal of Western beauty standards, contributing to body dissatisfaction and the desire for cosmetic enhancements (82).

The significance of this study lies in its potential to provide insights into how cultural and social dynamics shape the motivations behind cosmetic surgery decisions among Kuwaiti youth. By understanding the interplay between social media influence, cultural expectations, and individual psychological factors, this research can inform public health strategies and educational interventions aimed at promoting healthy body image and self-esteem among young individuals (83). Furthermore, the findings can contribute to the development of culturally sensitive policies that address the unique challenges faced by Kuwaiti youth in navigating the pressures of cosmetic surgery in a rapidly evolving social media landscape (84, 85).

The concept of perceived behavioral control also plays a crucial role in the decision-making process regarding cosmetic surgery. The study indicates that individuals who feel empowered by having access to credible information and reputable practitioners are more likely to consider cosmetic procedures (27, 80). This suggests that social media can serve as a double-edged sword; while it can provide valuable information that aids informed decision-making, it can also perpetuate harmful beauty standards that pressure individuals into making choices that may not align with their true desires or well-being (27, 80).

In conclusion, the influence of social media on cosmetic surgery intentions in Kuwait is shaped by a complex interplay of cultural norms, the power of influencers, and individual perceptions of control (86, 87). As social media continues to evolve, its role in shaping beauty standards and influencing personal decisions regarding cosmetic surgery will likely remain significant, necessitating ongoing research and awareness of its psychological impacts.

The findings have significant implications for healthcare providers and policymakers in Kuwait. Healthcare providers should consider the pervasive influence of social media when advising patients on cosmetic surgery. Providing patients with accurate information and realistic expectations is crucial in counteracting the sometimes exaggerated or idealized representations of cosmetic procedures found on social media. Moreover, policymakers should consider regulating the portrayal of cosmetic surgery on social media platforms to ensure that advertisements and influencer endorsements are responsible and transparent.

Given the pervasive influence of social media on cosmetic surgery intentions, healthcare providers should consider implementing targeted digital health interventions. These could include educational campaigns on social media platforms to promote realistic beauty standards and provide accurate information about the risks and benefits of cosmetic procedures. Collaborations with influencers who advocate for body positivity and ethical advertising practices can also help counteract misleading portrayals of cosmetic surgery. Additionally, healthcare practitioners can utilize social media to enhance digital health literacy, enabling individuals to critically evaluate cosmetic surgery content online. By adopting these strategies, healthcare providers can play a proactive role in mitigating the negative impacts of social media on cosmetic surgery decisions.

## 6 Conclusion

This study sheds light on how social media influences young Kuwaiti individuals’ views and intentions toward cosmetic facial surgery. By utilizing the Theory of Planned Behavior (TPB), the findings reveal that subjective norms, influenced by social media platforms, play a pivotal role in shaping cosmetic surgery intentions. Additionally, attitudes and perceived behavioral control significantly contribute to individuals’ decision-making processes, highlighting the complex interplay between personal beliefs and societal pressures. The study's findings have important theoretical and practical implications for healthcare practitioners, policymakers, and educators. Healthcare professionals should develop educational programs that promote realistic expectations and provide accurate information about cosmetic procedures. Policymakers should consider regulating social media advertising related to cosmetic surgery to ensure transparent and ethical marketing practices. Meanwhile, educators should promote digital literacy, enabling young individuals to critically evaluate beauty standards portrayed on social media and fostering healthier body image perceptions.

### 6.1 Limitations

Despite the contributions of this study, several limitations must be acknowledged. First, the study primarily relied on self-reported data, which may be subject to social desirability bias. Future studies could incorporate qualitative methods such as in-depth interviews to gain richer insights into psychological motivations and cultural perceptions influencing cosmetic surgery decisions.

Second, the cross-sectional design limits the ability to infer causal relationships between social media exposure and cosmetic surgery intentions. Future research could employ longitudinal studies to track how these influences evolve over time.

Third, the study sample was predominantly female (84.9%), limiting the generalizability of findings to male participants. Future research should aim for more balanced gender representation to explore potential gender differences in how social media influences attitudes toward cosmetic surgery.

Fourth, while this study focused on social media influences, other sociocultural factors—such as family expectations, religious beliefs, and psychological well-being—may also play a role in shaping cosmetic surgery decisions. Future research should integrate a more holistic framework to examine how these factors interact with digital media exposure.

Lastly, technological advancements in augmented reality (AR) filters and AI-generated beauty standards may further exacerbate body image concerns. Future studies should explore the role of AI-driven beauty enhancement tools in influencing individuals’ attitudes and intentions toward cosmetic procedures.

## Data Availability

The raw data supporting the conclusions of this article will be made available by the authors, without undue reservation.
