# Supplementary material for: Influence of social media on cosmetic facial surgeries among individuals in Kuwait: employing the theory of planned behavior
Source: Front Digit Health. 2025 Apr 24;7:1546128. doi: 10.3389/fdgth.2025.1546128 (PMC12058905; doi:10.3389/fdgth.2025.1546128)
Supplement: Supplementary file 1 [file Datasheet1.pdf]

SUPPLEMENTARY MATERIAL

Questionnaire

Demographic Information

Gender	Age group
Nationality	occupation
Education	Martial State

General Attitude Towards Cosmetic Surgery (MCQ)

Have you ever undergone any form of cosmetic facial surgery?

A) Yes, I have undergone cosmetic facial surgery.
B) Yes, but it was for medical rather than cosmetic reasons.
C) No, but I am considering it for the future.
D) No, and I do not plan to undergo any cosmetic facial surgery.

If you have undergone or are considering cosmetic facial surgery, what is the primary reason for your decision?

A) To enhance my self-esteem and confidence.

B) To correct a feature, I am not satisfied.

C) Influenced by social media or celebrity culture.

D) Medical reasons (e.g., injury or health conditions).

E) Maintaining social or professional expectations.

F) Other (please specify).

G) Not applicable/I did not consider cosmetic facial surgery.

Influence of Social Media (MCQ)

Which social media app do you use the most?

Facebook	Instagram
Twitter	TikTok
Snapchat	Other (Please specify)

How much time do you typically spend on this app each day?

Less than 30 minutes	2 to 3 hours
30 minutes to 1 hour	3 to 4 hours

1 to 2 hours	More than 4 hours

e-WOM (Likert (SD/SA)

e-WOM1: When I seek information about cosmetic facial surgeries, I explore the opinions of other individuals on social media platforms.

e-**WOM2**: The opinions of other individuals on social media platforms influence my consideration of cosmetic facial surgery.

e-WOM3: I read social media posts and reviews about cosmetic facial surgeries to understand whether they leave a positive impression on others, considering such procedures.

e-WOM4: Before considering cosmetic facial surgery, I read online reviews and testimonials from others on social media to ensure that my decision was well-informed.

e-WOM5: I consult online (through social networks, forums, blogs, etc.) with other individuals to help choose the right practitioner or clinic for cosmetic facial surgeries.

e-WOM6: I gathered information from other individuals' reviews on social media before deciding on cosmetic facial surgery.

e-WOM7: I am concerned about my decision to undergo cosmetic facial surgery if I do not read online reviews and testimonials from others.

e-WOM8: When considering cosmetic facial surgery, online reviews and testimonials from others make me feel confident about my decision.

Attitude Towards Cosmetic Facial Surgery (Employing Theory of Planned Behavior) (Likert (SD/SA)

ATT1: My awareness of social media trends impacts my consideration of undergoing cosmetic facial surgeries.

ATT2: Positive beliefs regarding the outcomes of cosmetic facial surgery influenced by social media affect my consideration for undergoing such surgery.

ATT3: My feelings towards the portrayals of cosmetic facial surgery on social media impact my consideration for undergoing cosmetic facial surgery.

ATT4: My engagement with social media content related to cosmetic facial surgery influences my attitude towards the intention to undergo cosmetic facial surgery.

ATT5: "Seeking information on cosmetic facial surgeries on social media is advantageous.

ATT6: Using social media to inform oneself about cosmetic facial surgery options is wise.

ATT7: Finding information on cosmetic facial surgeries on social media is a pleasant action.

ATT8: Looking up cosmetic facial surgery information on social media is attractive.

Influencer Marketing (Likert (SD/SA)

IM1: When I seek information about cosmetic facial surgeries, I look for the opinions of influencers on social media platforms.

IM2: The opinions of influencers on social media platforms influenced my consideration of undergoing cosmetic facial surgery.

Content Marketing (Likert (SD/SA)

CM1: When I seek information about cosmetic facial surgeries, I look for content from reputable clinics and brands on social media.

CM2: The content provided by cosmetic surgery clinics and brands is valuable and informative regarding procedures and outcomes.

CM3: Content from cosmetic surgery clinics and brands on social media explains the benefits of these surgeries.

CM4: The content of cosmetic facial surgeries on social media affects my consideration of undergoing such surgeries.

Subjective Norms (Likert (SD/SA)

SN1: Behavioral expectations from friends, family, other consumers, and influencers on social media impact my consideration of undergoing cosmetic facial surgeries.

SN2: Social pressure from friends, family, and influencers on social media affects my intention to consider cosmetic facial surgery.

SN3: Social approval, influenced by social media, positively impacts my intention to undergo cosmetic facial surgery to convey a positive impression within my social circles.

SN4: People who are important to me think I should consider reputable sources and opinions on social media when deciding to undergo cosmetic facial surgeries.

SN5: Most people who are important to me would want me to consider expert and influencer opinions on social media regarding cosmetic facial surgery when making decisions.

SN6: People whose opinions I value would prefer to consult and value professional advice and testimonials on social media about cosmetic facial surgeries before making a decision.

Health Consciousness (Likert (SD/SA)

HC1: I think of myself as a consumer who is conscious of aesthetic and cosmetic health.

HC2: I often think about cosmetic health issues and the impact of aesthetic procedures.

HC3: I plan to consider cosmetic facial surgery in the future, influenced by the information and trends I observe on social media.

Perceived Behavioral Control (Likert (SD/SA)

PBC1: I am confident that if I want, I can consult social media to make an informed decision about undergoing cosmetic facial surgery."

PBC2: I can use social media to obtain reliable information and advice on cosmetic facial surgery.

PBC3: I have enough resources (e.g., access to reputable social media accounts and platforms) to make informed decisions about cosmetic facial surgery.

PBC4: I have enough time to research and evaluate information on cosmetic facial surgery through social media before making decisions.

Intention (Likert (SD/SA)

INT1: I am willing to consider cosmetic facial surgery because I believe it will be beneficial to my personal wellbeing.

INT2: I am willing to consider cosmetic facial surgery because I trust that reputable practitioners ensure that the procedures are safe with minimal risk.

INT3: I am planning to recommend clinics or practitioners who are well reviewed on social media for cosmetic facial surgery when someone asks me for advice in the future.

INT4: I intend to suggest considering social media testimonials and reviews about cosmetic facial surgeries when someone is seeking advice on this matter in the future.

INT5: I will expend efforts to persuade everyone who asks me about cosmetic facial surgery to consider reputable sources and influencers' recommendations on social media in the future.
